# Prediction of early prognosis after traumatic brain injury by multifactor model

**DOI:** 10.1111/cns.13935

**Published:** 2022-08-26

**Authors:** Bocheng Yang, Xiaochuan Sun, Quanhong Shi, Wei Dan, Yan Zhan, Dinghao Zheng, Yulong Xia, Yanfeng Xie, Li Jiang

**Affiliations:** ^1^ Department of Neurosurgery the First Affiliated Hospital of Chongqing Medical University Chongqing China

**Keywords:** age, apolipoprotein E (APOE), C‐reactive protein (CRP), glasgow coma scale (GCS), interleukin‐8 (IL‐8), Marshall CT score, traumatic brain injury (TBI)

## Abstract

**Aims:**

To design a model to predict the early prognosis of patients with traumatic brain injury (TBI) based on parameters that can be quickly obtained in emergency conditions from medical history, physical examination, and supplementary examinations.

**Methods:**

The medical records of TBI patients who were hospitalized in two medical institutions between June 2015 and June 2021 were collected and analyzed. Patients were divided into the training set, validation set, and testing set. The possible predictive indicators were screened after analyzing the data of patients in the training set. Then prediction models were found based on the possible predictive indicators in the training set. Data of patients in the validation set and the testing set was provided to validate the predictive values of the models.

**Results:**

Age, Glasgow coma scale score, Apolipoprotein E genotype, damage area, serum C‐reactive protein, and interleukin‐8 (IL‐8) levels, and Marshall computed tomography score were found associated with early prognosis of TBI patients. The accuracy of the early prognosis prediction model (EPPM) was 80%, and the sensitivity and specificity of the EPPM were 78.8% and 80.8% in the training set. The accuracy of the EPPM was 79%, and the sensitivity and specificity of the EPPM were 66.7% and 86.2% in the validation set. The accuracy of the early EPPM was 69.1%, and the sensitivity and specificity of the EPPM were 67.9% and 77.8% in the testing set.

**Conclusion:**

Prediction models integrating general information, clinical manifestations, and auxiliary examination results may provide a reliable and rapid method to evaluate and predict the early prognosis of TBI patients.

## Graphical abstract

By analyzing the admission information and examination results of patients with traumatic brain injury (TBI), the factors that may affect the early prognosis of patients with TBI were obtained. Then these factors are combined by mathematics to establish a prediction model to implement the effective prediction of early prognosis of patients with TBI. The results show that this idea is feasible.

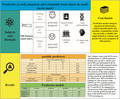

## 1 INTRODUCTION

Traumatic brain injury (TBI) is one of the most common emergency conditions in neurosurgery and is becoming the leading cause of disability and death in all types of traumatic diseases all over the world.^1^, ^2^ Previous studies have shown that more than 50 million people around the world suffer from TBI every year,^3^ becoming a serious burden to families and society.^4^, ^5^, ^6^ Although significant efforts have been made in the prevention and treatment of TBI, the progress in the improvement of TBI prognosis is still far less than satisfactory.^7^ An effective and reliable prognosis prediction after TBI is crucial for making the optimal treatment plan and supporting anxious family members and caregivers so that they can manage the situation and make decisions. However, the process after TBI is complicated and variable, involving a lot of risk factors that may influence the outcome of TBI. Some factors like systolic blood pressure (SBP), abnormal pupillary response, Glasgow coma scale (GCS), and computed tomography (CT) findings have been investigated in many studies, while other factors like some kind of genes and laboratory values which attract less attention may also relate to the process after TBI. Therefore, how accurately predicting the prognosis of TBI is still a challenge.

Although a variety of prediction models have been designed to predict the prognosis and mortality of patients with TBI, only 37% or less of the medical staff believe that the prediction models and artificial intelligence have achieved their design purpose and can make effective predictions.^8^, ^9^, ^10^ Therefore, a prediction model trusted by most medical workers to effectively predict the disease development of patients with TBI is still needed at the present.

This study aims to explore a reliable model to predict the early prognosis of patients with TBI. To achieve this aim, comprehensive factors and parameters that can be quickly obtained in emergency work from medical history, physical examination, and supplementary examinations were included and analyzed, then the models with different factors and parameters were tested by comparing with the early prognosis of TBI patients.

## 2 SUBJECTS AND METHODS

### 2.1 Subjects

This study was approved by the Ethics Committee of the First Affiliated Hospital of Chongqing Medical University. The model was designed based on the relevant medical records of patients diagnosed with TBI who were hospitalized in the First Affiliated Hospital of Chongqing Medical University and University‐Town Hospital of Chongqing Medical University between June 2015 and June 2021. The use of non‐patient privacy medical records in the study was fully explained to the patient or legal representative during the patient's admission, and informed consent and full written authorization were obtained.

The inclusion criteria for TBI patients in this study were as follows: (1) patients who had a clear history of TBI, (2) patients aged 15–75 years old, (3) patients who had been admitted to the hospital within 8 h after injury. The exclusion criteria were as follows: (1) lack of relevant medical records, (2) patients who had clear indications and no absolute contraindications for surgery, but had not undergone surgery, (3) patients discharged within 24 h, (4) patients with multiple injuries or concomitant serious life‐threatening organ or system diseases, such as severe heart disease, tumor, etc. All patients received treatment according to their condition under the guidance of the latest TBI guideline after admission.

### 2.2 Model construction and testing

The main purpose of this study is to predict the prognosis of patients at the early stage (14 days after TBI) of TBI^11^, ^12^, ^13^ by an early prediction prognosis model(EPPM). All patients enrolled in this study in the First Affiliated Hospital of Chongqing Medical University were then divided into the training set and validation set. And patients collected from the University Town Hospital were taken as a testing set. All patients in each set were divided into the good prognosis group and the poor prognosis group according to the early prognosis of patients. The standards of good prognosis group included: (1) patients showed improvement in consciousness, speech function, or physical activity. (2) patients showed no serious complications. (3) The Modified Rankin Scale (MRS) score of patients less than or equal to 3. While the standards of the poor prognosis group included: (1) the patients' consciousness, language function, and body movement deteriorated or did not improve significantly. (2) The patient developed life‐threatening complications during hospitalization, such as a severe infection, shock, etc. (3) The patient was dead. The whole process of data collection and prognostic judgment were made by two neurosurgeons with more than 20 years of clinical experience.

#### 2.2.1 Screening of predictive indicators

Patients in the training set were randomly divided into two parts in proportion (part1: part2 ≈ 1:2). All clinical data of patients in part1 were analyzed, including general clinical information, laboratory results, and CT image. Clinical data between the poor prognosis group and the good prognosis group would be compared in part1. Then the clinical data with significant statistical differences between groups were obtained. Multivariate logistic regression analysis was used as a further test to screen out the clinical data that may lead to the poor early prognosis of patients in the early stage of TBI. All the clinical data screened by the test were counted as the predictive indicators of the EPPM.

#### 2.2.2 Model construction, training, and chosen

The main structure of this model is a scoring system, which grades and scores the determined predictive indicators. All patients are scored according to the specific situation of each clinical data, and the sum of the scores is taken as the final score of patients. Determining a threshold for the total score will help distinguish the boundary between good and poor prognosis in the early stage of TBI.

The training of the EPPM was initiated to fine‐tune the specific grades and scores of each predictor. This step will be achieved by integrating all patients' clinical data in part2 into EPPM for prediction. We drew the ROC curve (receiver operating characteristic curve) of the prediction model and calculated the AUC (area under curve) after each adjustment. Then the optimal threshold was found based on the ROC, and the prediction accuracy, specificity, and sensitivity of the model under the optimal threshold were calculated. The study team would jointly determine the appropriate scoring scheme and design the prediction system. Decision‐making was based on the result of comparing the model prediction accuracy, specificity, and sensitivity under the optimal threshold of each adjustment comprehensively.

#### 2.2.3 Model verification and testing

The validation set and test set patient data were integrated into the EPPM to validate and test the predictive effectiveness of the EPPM. This step also needs to draw ROC curves and calculated AUC. The prediction accuracy, specificity, and sensitivity of EPPM at the optimal threshold determined from the training set were calculated to evaluate the EPPM. If the model verification and testing results meet the team's expectations, the final model will be determined; otherwise, the second step (model construction, training, and chosen) would be taken.

### 2.3 Data processing and analysis

All statistical processes and data analysis involved in this study were performed by SPSS software (version 25.0 for Windows, IBM Corp.,) and GraphPad Prism (version 8.0 for Windows, GraphPad Software,). The Shapiro–Wilk test was used to determine whether the measurement data were normally distributed and the homogeneity of variance test was carried out. Measurement data were presented as mean ± standard deviation, and the student's *t*‐test or Mann–Whitney was used to test the difference in measurement data between the groups with good and with poor early prognosis. Chi‐square (χ^2^) tests or Fischer's exact test was used to compare the difference of clinical data and other categorical data between the two groups of patients, and were presented as numbers and percentages. Multivariate logistic regression analysis would be used to examine possible independent risk factors. *p* < 0.05 was considered statistically significant. The ROC curve was drawn and AUC was calculated to evaluate the effectiveness of the prediction model. AUC >0.60 was considered better prediction ability.

## 3 RESULT

A total of 675 patients were recruited for this study, and 43 patients were excluded according to the exclusion criteria. Finally, a total of 632 consecutive patients were included in this study and were randomly divided into 3 sets: training set (part1: 168 patients, part2: 300 patients), validation set (100 patients), and testing set (64 patients).

### 3.1 Training set

#### 3.1.1 General clinical data

As shown in Table 1, among the general clinical data, age, damage area, APOE gene, and GCS score may be related to poor early prognosis. The rate of early poor prognosis for patients beyond 45 years old was 48%, significantly higher than that for patients under 45 years old (44%, *p* < 0.05 by Chi‐square tests). The early poor prognosis rate of TBI patients with GCS scores ≤8 was 69%, which was significantly higher than those with GCS scores >8 (37%, *p* < 0.001 by Chi‐square tests). However, no significant statistical difference in the rate of early poor prognosis was found among different injury mechanisms (*p* > 0.05 by Chi‐square tests), and the differences in brain damage area may be associated with early prognosis of TBI (*p* < 0.001 by Chi‐square tests). Meanwhile, patients with or without hypertension, diabetes, the habit of smoking, and alcohol also did not show a significant difference in the early poor prognosis (*p* > 0.05 by Chi‐square tests). Therefore, age, damage area, APOE gene, and GCS score were included as predictors for predicting early poor prognosis in the prediction models.

**TABLE 1:** The General clinical data of the 168 patients in the training set

Items	Early poor prognosis rate(%)		*p*
	Yes	Not	
Male (91)	48 (44/91)	44 (34/77)	0.58
Age>45 (98)	54 (53/98)	36 (25/70)/	0.03
Mechanism of injury			0.93
Traffic injury (38)	50 (19/38)	/	
Striking injury (35)	46 (16/35)	/	
Fall from height (53)	48 (25/53)	/	
Others (42)	43 (18/42)	/	
Damaged area			0.002
Frontal lobe	60 (47/78)	/	
Temporal lobe	44 (19/43)	/	
Parietal lobe	23 (5/22)	/	
Occipital lobe	28 (7/25)	/	
Personal history			
Smoking (75)	48 (36/75)	45 (42/93)	0.71
Alcohol‐drinking(89)	46 (41/89)	47 (37/79)	0.92
Past medical history			
Hypertension (78)	49 (38/78)	44 (40/90)	0.57
Diabetes (70)	44 (31/70)	48 (47/98)	0.64
GCS Score			
≤8 (49)	69 (34/49)	37 (44/119)	<0.001
9–12 (86)	45 (39/86)	/	
13–15 (33)	15 (5/33)	/	
APOE Gene			
ε4‐carriers (65)	49 (37/65)	45 (46/103)	0.022

Abbreviations: APOE, Apolipoprotein E; Damaged area: the major injured lobes determined by clinician analysis; GCS Score, Glasgow coma scale.

#### 3.1.2 Laboratory results data

The commonly used laboratory results data of 168 patients with early poor and good prognoses were also collected and analyzed, and no significant differences were found among most laboratory results data, such as hemoglobin, platelets, electrolyte, albumin, etc (*p* > 0.05 by Chi‐square tests). However, the C‐reactive protein (CRP) level in the serum of patients in the poor prognosis group was 14.20 ± 5.73 mg/L, which was significantly higher than that in the good prognosis group (11.97 ± 3.84 mg/L, *p* < 0.05 by Student's *t*‐test). And the level of interleukin‐8 (IL‐8) in the poor prognosis group was 23.68 ± 5.35 pg/mL, which was also significantly higher than that in the good prognosis group (21.59 ± 4.10 pg/mL, *p* < 0.05 by Student's t‐test, Table 2). Therefore, CRP and IL‐8 were included as predictors in the prediction models.

**TABLE 2:** The Laboratory results of the 168 patients in the training set

Items	Early prognosis		*p*
	Poor	Good	
Hemoglobin (g/L)	112.00 ± 27.20	116.70 ± 18.51	0.22
WBC (10^9)	11.63 ± 3.61	12.05 ± 3.72	0.16
Albumin (g/L)	37.83 ± 5.81	35.91 ± 6.40	0.23
APTT (s)	38.11 ± 6.31	36.24 ± 7.26	0.15
PT (s)	18.46 ± 4.12	18.91 ± 5.11	0.42
INR	1.42 ± 0.31	1.32 ± 0.40	0.21
CRP (mg/L)	14.20 ± 5.73	11.97 ± 3.84	0.01
PCT (ng/L)	0.62 ± 0.41	0.59 ± 0.36	0.18
Interleukin‐6 (pg/ml)	7.53 ± 2.35	6.97 ± 1.29	0.06
Interleukin‐8 (pg/ml)	23.68 ± 5.35	21.59 ± 4.10	0.01
TB (μmol/L)	5.9 ± 1.6	6.7 ± 1.8	0.21
DB (μmol/L)	3.5 ± 1.6	3.4 ± 1.3	0.11
ALT (U/L)	30.1 ± 15.3	32.4 ± 18.3	0.31
AST (U/L)	33.2 ± 16.5	34.5 ± 11.2	0.11
K+ (mmol/L)	3.8 ± 1.2	4.0 ± 1.1	0.13
Na + (mmol/L)	141 ± 13.5	139 ± 15.2	0.16
Ca_2_ (mmol/L)+	2.34 ± 0.36	2.26 ± 0.41	0.09
Creatinine (μmol/L)	80.12 ± 17.46	71.58 ± 13.05	0.28
Cholesterol (mmol/L)	4.07 ± 1.85	4.28 ± 2.32	0.15

Abbreviations: APTT, Activated partial thromboplastin time; CRP, C‐reactive protein; DB, Direct bilirubin; PCT, procalcitonin; PT, Prothrombin time; TB, Total bilirubin; WBC, White blood cells.

#### 3.1.3 CT image results

Marshall CT score system was performed to evaluate the CT manifestation of patients. The poor prognosis rate (65.0%) of TBI patients with Marshall CT score ≥ IV was significantly higher than that of<IV (29.5%, *p* < 0.05 by Chi‐square tests) (Table 3). So we considered the Marshall CT score as a predictor of early poor prognosis in the models.

**TABLE 3:** Comparison of the CT image results of the 168 patients in the training set

	Early poor prognosis rate(%)	*p*
Marshall CT Class		/
I(18)	16.7(3/18)	/
II(32)	25.0(8/32)	/
III(38)	39.5(15/38)	/
IV(27)	48.1(13/27)	/
V(28)	71.4(20/28)	/
VI(25)	76.0(19/25)	/
Marshall CT Class		<0.001
≥IV	65.0(52/80)	/
<IV	29.5(26/88)	/

*Note*: Marshall CT Class I, normal CT; II, cisterns present, shift<5 mm; III, cisterns compressed, shift<5 mm; IV, shift>5 mm, V, evacuated mass; VI, non evacuated mass.

#### 3.1.4 Multivariate logistic regression analysis

Multi‐factor logistic regression analysis was used to eliminate the interaction of risk factors. The results suggest that age (*p* < 0.001, OR = 2.833, 95% CI = 1.637–4.904), damage area(*p* = 0.001, OR = 2.006, 95%CI = 1.321–3.045), APOE gene(*p* = 0.015, OR = 3.247, 95% CI = 1.131–9.318), GCS score(*p* < 0.001, OR = 3.612, 95% CI = 1.770–7.374), CRP(*p* = 0.002, OR = 2.415, 95% CI = 1.378–4.232), IL‐8(*p* = 0.034, OR = 2.767, 95% CI = 1.082–7.079), and Marshall CT score(*p* < 0.001, OR = 2.617, 95% CI = 1.487–2.734) may be independent risk factors for early prognosis of TBI patients (Table 4).

**TABLE 4:** The General information of the 168 patients in the training set

Items	Early prognosis		Logistic regression analysis	
	Poor	Good	*p*	*OR* (CI_95_)
Age			<0.001	2.833 (1.637–4.904)
≥60	34	15		
46–59	20	31		
≤45	24	44		
Damaged area			0.001	2.006 (1.321–3.045)
frontal lobe	47	31		
temporal lobe	19	24		
parietal lobe	5	17		
occipital lobe	7	18		
GCS Score			<0.001	3.612 (1.770–7.374)
≤8	34	15		
9–12	36	53		
13–15	8	22		
ε4‐carriers	37	28	0.019	2.970 (1.194–7.391)
CRP			0.002	2.415 (1.378–4.232)
≥15	33	17		
10–15	35	48		
<10	10	25		
IL‐‐8			0.034	2.767(1.082–7.079)
≥20	61	55		
10–20	17	35		
<10	0	0		
Marshall CT Class			<0.001	2.617 (1.487–2.734)
I	3	15		
II	8	24		
III	15	23		
IV	13	14		
V	20	8		
VI	19	6		

#### 3.1.5 Specificity and sensitivity of EPPM

Age, damage area, APOE gene, GCS score, CRP, IL‐8, and Marshall CT score were finally incorporated into the model as predictors of early poor prognosis in patients with TBI. The design scoring rules were established as shown in the table after repeated calculations by the team (Table 5). (All the predictors of a patient were calculated into different scores according to their degree.)

**TABLE 5:** Grading rules^*^

Items	0	1	2	3	4	5	6
Age					≤45	46–59	≥60
Damaged area			Parietal/occipital	temporal	frontal		
GCS Score		13–15		9–12			≤8
ε4‐carriers	NO		YES				
CRP			<10	10–15		≥15	
IL‐‐8	<10		10–20		≥20		
Marshall CT Class		I/II			III/IV		V/VI

Grading rules^*^: All the predictors of a patient are calculated into different scores according to their degree. The total score is the sum of the scores of each predictor.

Example: A patient (46 years old, temporal lobe damage, GCS score: 8, ε4‐carriers, CRP: 14.2 mg/L, IL‐8: 15.20 pg/mL, Marshall CT Class: III), scores of each predictor (Age: 5, Damaged area: 3, ε4‐carriers: 2, CRP:3, IL‐8: 2, Marshall CT Class: 4), the total score: 5 + 3 + 2 + 3 + 2 + 4 = 19.

**FIGURE 1:**
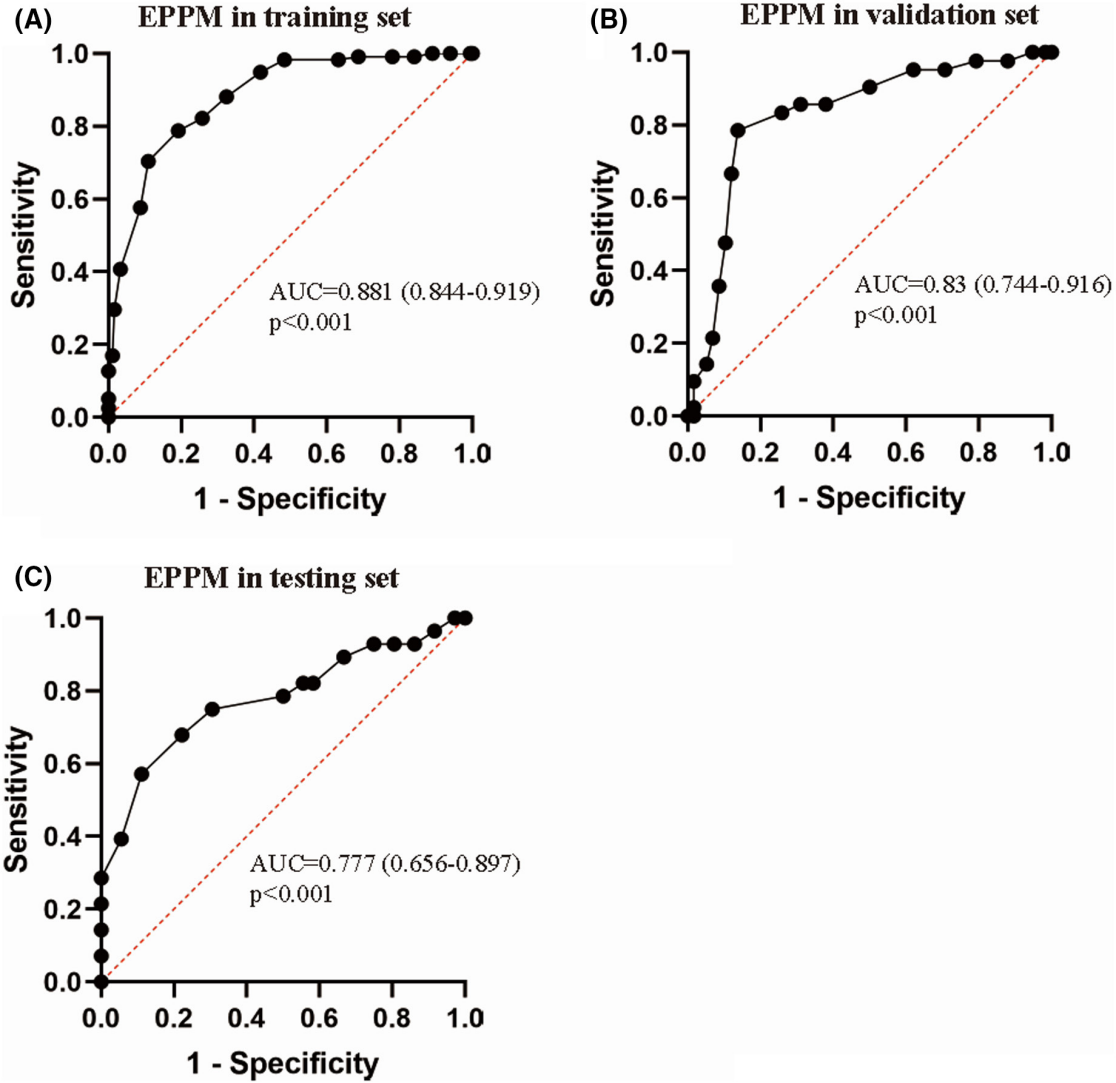
The ROC curve (receiver operating characteristic curve) of EPPM (early prognosis prediction model) in the training set, validation set, and testing set. A: The AUC(area under curve) of the ROC curve in the training set was 0.881 (0.844–0.919) (*p* < 0.001). B: The AUC of the ROC curve in the validation set was 0.830 (0.744–0.916) (*p* < 0.001). C: The AUC of the ROC curve in the validation set was 0.777 (0.656–0.897) (*p* < 0.001)

The EPPM of patients with TBI was built based on the scoring system. The ROC curve was drawn and AUC was calculated to evaluate the prediction ability of the EPPM (Figure 1). The result showed that the AUC of EPPM was 0.881 (0.844–0.919)(*p* < 0.001). The comprehensive prediction efficiency of the model is the best when the threshold was 23.5. The sensitivity and specificity of the EPPM were 78.8% and 80.8%, and the accuracy of the EPPM was 80%.

### 3.2 Validation set

In order to test and verify the predictive ability of the EEPM in this medical center, we included the data of validation set into the EPPM for testing. The results showed that the accuracy of the EPPM was 79%, and the sensitivity and specificity of the EPPM were 66.7% and 86.2%, when the threshold was 23.5. The AUC of the ROC curve was 0.830 (0.744–0.916) (*p* < 0.001) (Figure 1).

### 3.3 Testing set

In order to test the generalization ability of the EPPM, we included the data of the testing set into the EPPM for testing. The results showed that the accuracy of the EPPM was 69.1%, and the sensitivity and specificity of the EPPM were 67.9% and 77.8%, when the threshold was 23.5. The AUC of the ROC curve was 0.777 (0.656–0.897) (*p* < 0.001) (Figure 1).

## 4 DISCUSSION

The main finding of this study was that, models based on comprehensive factors and parameters from medical history, physical examination, and supplementary examinations could provide an accurate and reliable prediction of the early prognosis of TBI patients. However, most of the general clinical data and laboratory results included in this study have very limited predictive value for patient prognosis. Compared with the previous CRASH model and IMPACT model, APOE, CRP, and IL‐8 were added to the prediction models designed in this study, which increased the value of these prediction models in predicting the prognosis.^11^, ^12^, ^14^

The influence of the location of brain injury on the early prognosis of patients with TBI was analyzed in this study. The results showed that the patients with a frontal lobe or temporal lobe injury were more likely to have a poor prognosis in the early stage of TBI. Most of the patients with poor prognoses showed deficits in cognitive function, which is similar to the results of previous clinical studies.^15^, ^16^ Some studies^16^, ^17^ showed that damage to the prefrontal cortex disrupts a variety of cognitive functions, including planning, problem solving, and time organization. And extensive lesions in the hippocampus and medial temporal cortex resulted in severe impairment of memory function.^18^ So the location of the damaged area in the brain may be crucial to determining the possible poor prognosis of early TBI patients, especially the possible cognitive deficiency.

As shown by the results of the study, APOE ε4 carriers were more likely to have early adverse outcomes of TBI than that non‐carriers, which is consistent with previous studies. APOE gene is one of the most studied genes that might be involved in the process after TBI. Our previous studies have shown that APOEε4 carriers might be apt to the clinical deterioration after TBI,^19^, ^20^ and APOEε4 carriers might have worse brain oxygen metabolism and more slow‐wave activities in electroencephalogram as compared to APOEε4 non‐carriers after TBI..^21^, ^22^ Other studies also showed that the carriers of the APOE ε4 allele had longer hospital stays and lower GOS scores and were more likely to have neurobehavioral disorders after TBI.^23^, ^24^ Meanwhile, APOE gene polymorphism is considered to be involved in a series of pathophysiological processes, which may interfere with the protection process and aggravate the injury process after TBI.^25^, ^26^, ^27^ Previously, the results of our studies have also shown that the APOEε4 might influence the balance of intracellular ion levels, inflammatory response, and blood–brain barrier permeability, which might influence the process of apoptosis and repair after TBI.^28^, ^29^, ^30^, ^31^, ^32^

The EPPM has taken the role of IL‐8 into account, and IL‐8 may have a negative impact on the prognosis in the early stage of TBI patients. Previous studies on TBI patients showed a close relationship between the elevated levels of IL‐8 in the cerebrospinal fluid and greater mortality risk,^33^ and severe TBI survivors had significantly lower serum IL‐8 levels at admission as compared with dead patients.^34^ This may be related to multiple inflammatory reactions involving IL‐8.^35^, ^36^ Moreover, IL‐8 may also accelerate the conversion of subdural effusion to a subdural hematoma, indirectly causing adverse effects on brain tissue.^37^ CRP that was included in the prediction models of the study is a sign of acute inflammatory response and is often elevated after infection or tissue damage.^38^ The significantly increased serum CRP level has been considered a strong indicator of secondary brain injury.^39^ Previous studies have shown that CRP levels are associated with severity and mortality in patients with TBI.^40^, ^41^ Meanwhile, in subarachnoid hemorrhage, intracerebral hemorrhage, and other brain injuries, CRP has also been found to be associated with a poorer prognosis of the diseases^42^, ^43^, ^44^ Therefore, both IL‐8 and CRP were included in the predictive models of this study, which may help to improve the accuracy of prediction models.

Marshall CT score involved in this study is one of the most used CT‐based scoring systems. Patients with a higher score may have a poor prognosis, which may be related to the extent of brain tissue damage reflected by imaging.^45^, ^46^ The results of this study also showed that the score may be effective for predicting the prognosis of patients with TBI. The general clinical data of hospitalized patients with TBI are also important information. Age and GCS scores were found to be related to the prognosis of TBI in the study. Previous studies have shown that age might be associated with the recovery and incidence of complications in TBI patients.^21^, ^22^, ^23^ GCS score is usually used for evaluating the severity of TBI, some literature has agreed that higher mortality rates were associated with lower admission GCS scores.^13^, ^47^ Therefore, the GCS score was considered to be an effective predictor of the prognosis of TBI patients by previous studies as well.^48^, ^49^

Although the results of this study are promising, it does have some limitations. First, the study was limited by the single center and small sample size which might lead to results bias. Second, more valuable observations indicator should be considered to improve the accuracy of predictions. Indicators such as EEG, CTP, and cerebral oxygen saturation, which may be related to the prognosis of TBI,^21^, ^22^, ^29^ should be incorporated into the prediction models appropriately. In addition, to improve the construction and efficiency of prediction models, machine learning will be the most concern in our further research.

## 5 CONCLUSION

Models integrating general information, clinical manifestations, and auxiliary examination results may provide a reliable and rapid prediction of the early prognosis of TBI patients. Furthermore, the involvement of some important indicators such as IL‐8 and APOE will increase the accuracy of the prediction models.

## AUTHOR CONTRIBUTIONS

All the authors contributed to the success of this research. This study was designed and managed by Yanfeng Xie and Li Jiang. Material preparation, data collection, and analysis were performed by Bocheng Yang, Yulong Xia, and Yanfeng Xie. The first draft of the manuscript was written by Bocheng Yang and Li Jiang. All authors made comments and suggestions on previous editions of the manuscript. The final manuscript was approved after all the authors had read it to their satisfaction.

## FUNDING INFORMATION

This study was funded by the National Natural Science Foundation for Youth of China (No. 81701226), Chongqing Medical Scientific Research Project (Joint project of Chongqing Health Commission and Science and Technology Bureau) (2022MSXM041) and Science and Technology Innovation Project of Chongqing University of Science and Technology (No. YKJCX2020834).

## CONFLICTS OF INTEREST

None of the authors of this article has a competing interest to declare.

## CONSENT TO PARTICIPATE

Informed consent was obtained from all study participants for this study.

## CONSENT FOR PUBLICATION

This research obtained written consent for publication from the patients or legal guardians of all participants in the research.

## CODE AVAILABILITY

Not applicable.

## Data Availability

Data supporting the findings of this research are available on request due to privacy/ethical restrictions.
